# Propensity-matched multicenter comparison of Parkinson’s disease outcomes with and without deep brain stimulation

**DOI:** 10.1038/s41531-025-01251-1

**Published:** 2026-01-08

**Authors:** Alireza Gharabaghi, Farzin Negahbani, Marius Keute

**Affiliations:** 1 Institute for Neuromodulation and Neurotechnology, University Hospital and University of Tübingen, 72076 Tübingen, Germany; 2 Center for Digital Health, 72076 Tübingen, Germany; 3 Center for Bionic Intelligence Tübingen Stuttgart (BITS), 72076 Tübingen, Germany; 4 German Center for Mental Health (DZPG), 72076 Tübingen, Germany; 5 Max Planck-University of Toronto Centre for Neural Science & Technology (MPUTC), Toronto (Canada), Tübingen, Germany

**Keywords:** Diseases, Medical research, Neurology, Neuroscience

## Abstract

Subthalamic deep brain stimulation is established for Parkinson’s disease, reducing funding for randomized trials and necessitating complementary approaches to assess outcomes in broader clinical contexts. Using publicly available multicenter data, we compared propensity-matched patients with and without stimulation. The intervention was associated with improved mid-term patient-reported motor and non-motor experiences of daily living, while ongoing follow-up will clarify long-term effects. This demonstrates feasibility of matched comparisons in open observational cohorts.

For individuals with Parkinson disease’s (PD), choosing between deep brain stimulation (DBS) and best medical therapy (BMT) raises important questions about how each option affects long-term motor function, cognition, and treatment burden^1–3^. Randomized controlled trials (RCTs) consistently demonstrate short-term benefits in motor function and quality of life^4,5^, but have limitations. Follow-up durations are often short, restricting long-term comparisons between DBS and BMT. In addition, RCT designs may introduce bias; patients assigned to BMT may experience negative expectations after not receiving surgery, which could influence outcomes^6,7^. In addition, PD progression differs between highly selected research cohorts and unselected clinic populations. Patients in routine care are often diagnosed and treated later and may have faster motor and cognitive decline than participants in clinical trials^8^. These differences highlight selection bias in research and the need for complementary approaches to improve external validity.

In parallel, once a therapeutic intervention becomes established, interest in conducting new RCTs typically declines, along with available funding. This is particularly true for DBS in PD, where efficacy for motor symptoms is well established. Yet many clinically relevant questions remain unresolved, including the long-term trajectory of treatment effects and the impact of DBS on non-motor symptoms. Addressing these gaps requires alternative study designs that can generate comparative evidence from observational cohorts. Although registries and open access research cohorts cannot replicate either the scientific rigor of RCTs or the full complexity of everyday practice, they provide a valuable resource for evaluating longer-term outcomes and comparing interventions in a broader clinical context^9,10^.

In this study, at approximately 1.7 years after baseline, the DBS group showed broad clinical benefits. Total MDS-UPDRS scores were significantly improved in the DBS group (*t* = −3.015, *p* = 0.004). Both clinician-rated Part I (*t* = −3.450, *p* = 0.002) and patient-reported Part I (*t* = −2.293, *p* = 0.025) showed a significant group difference favoring DBS. Patient-reported motor disability scores measured by Part II were also significantly reduced in the DBS group (*t* = −2.346, *p* = 0.025). Motor complications measured by Part IV were significantly lower as well (*t* = −2.869, *p* = 0.007). Motor examination scores (Part III) approached significance at the trend level (*t* = −1.913, *p* = 0.060). LEDD was numerically lower in the DBS group but was not statistically significant (*t* = −1.247, *p* = 0.217). No group differences were observed for MoCA or RBDQ.

At approximately 2.9 years after baseline, several of these differences persisted. Patient-reported non-motor experiences of daily living (Part I) remained significantly lower in the DBS group (*t* = −2.984, *p* = 0.008). Patient-reported motor disability scores (Part II) continued to favor DBS (*t* = −2.214, *p* = 0.034). Motor complications were again significantly reduced in the DBS group (*t* = −3.581, *p* = 0.002). Clinician-rated Part I (*t* = −1.943, *p* = 0.069) and total MDS-UPDRS (*t* = −1.944, *p* = 0.069) approached significance at the trend level, and motor examination scores (Part III) showed no between-group difference (*t* = 0.360, *p* = 0.721). LEDD remained numerically lower in the DBS group but was not statistically significant (*t* = −1.299, *p* = 0.203). MoCA and RBDQ again showed no evidence of group differences.

The effect sizes for key outcomes such as patient-reported motor and non-motor experiences of daily living and motor complications increased over time and were large, with d-values approaching or exceeding 1 in specific domains, indicating substantial and clinically meaningful separation between groups in favor of DBS (Table 2).

These analyses indicate that the cohort receiving DBS with medication experienced sustained advantages over the matched cohort receiving medication alone in several clinically relevant domains across the three-year observation period. Benefits were most pronounced in patient-reported motor (Part II) and non-motor experiences (Part I) of daily living, as well as in motor complications (Part IV), whereas gains in motor examination scores (Part III) diminished over time. Cognitive function and REM sleep behavior did not differ between groups at any interval. The pattern of results with strong baseline covariate balance and temporal comparability supports the feasibility of propensity score-matched comparisons in multicenter observational datasets and illustrates the trajectory of DBS-related effects across mid-term follow up outside traditional trial settings, consistent with prior matched cohort findings^11^. In contrast to earlier propensity-based studies that relied on administrative data and healthcare utilization variables as indirect proxies for disease severity^12,13^, the present analysis makes use of comprehensive clinical phenotyping and disease duration as matching covariates. This approach ensures closer alignment of symptom burden and disease stage across groups and enables comparison of clinically meaningful motor and non-motor domains. The reduced sample sizes at later timepoints primarily reflect irregular visit schedules and incomplete longitudinal data within the dataset, affecting generalizability of the present analysis. Continued follow-up will increase the number of evaluable cases and help clarify long-term effects.

Despite clinical multi-feature matching, residual confounding cannot be excluded, as DBS response may vary with individual disease characteristics such as motor phenotype or genetic background. Incorporating genotyping and more detailed phenotyping in future cohorts could help account for this biological heterogeneity and improve patient-specific treatment selection. Additionally, stimulation parameters, electrode configurations, or programming, which were not available in this data, may contribute to outcome variability. No significant group differences were observed for MoCA, or REM sleep behavior disorder symptoms, consistent with reports that non-motor effects of DBS vary with electrode location and baseline severity^14–16^. These null findings may also reflect limited sensitivity of the MoCA and RBDQ to subtle cognitive or sleep behavior changes, particularly in participants without marked baseline impairment. Future studies should incorporate more specific neuropsychological and polysomnographic assessments to capture these effects more accurately. Furthermore, the number of comparisons increases the risk of false positive findings. Clinically relevant and coherent patterns and effect sizes across time, such as the consistent advantages in patient-reported motor and non-motor experiences of daily living, are therefore more informative than isolated statistical values at individual timepoints.

Although observational and not causal, this analysis shows that propensity matching enhances comparability and that open multicenter datasets can yield reproducible comparative evidence. Expanding such resources with longer follow-up and integrated clinical, neuroimaging, genetic, and electrophysiological data will refine patient selection and guide individualized DBS management across disease progression.

## Methods

### Data source and study design

This study used data from the Parkinson Progression Markers Initiative (PPMI) to compare outcomes in PD with and without DBS. The goal was not to provide definitive treatment recommendations but to test the feasibility of propensity-matched comparisons using publicly available data. This proof-of-concept analysis may inform future studies as multicenter observational datasets expand.

### Study population

The PPMI dataset contains longitudinal data from 1061 individuals with PD, including 100 participants who underwent DBS (subthalamic nucleus (STN) = 62, globus pallidus internus (GPI) = 12, unknown targets = 26). For this analysis, we included those who received STN-DBS (48 bilateral, 7 left, 7 right; 39 males, 23 females). The remaining 961 non-DBS participants formed the pool for identifying matched comparators for follow-up analysis. PPMI does not provide stimulation parameters, electrode configurations, or programming data.

### Propensity score matching

Propensity score matching (PSM, MatchIt^17^ package implemented in R^18^) was applied to identify comparable patients without DBS based on age, disease duration since symptom onset, the Movement Disorder Society-Sponsored Revision of the Unified Parkinson’s Disease Rating Scale (MDS-UPDRS) Part I (rater report), I (patient report), II, III, and IV scores^19^. The propensity score model estimated, for all patients, the probability of receiving DBS based on their baseline characteristics, allowing the matching of DBS patients with clinically comparable BMT patients. Propensity score matching was applied to enhance baseline comparability rather than to establish causal effects. This design supports estimation of adjusted group differences within an observational framework but does not account for unmeasured confounding or treatment selection mechanisms. The objective was to test the feasibility and validity of this approach using open multicenter data, not to infer causal treatment efficacy. Matching used 1:1 nearest neighbor propensity score matching based on propensity scores from a logistic regression model.

### Assessment of covariate balance

Covariate balance was evaluated using standardized mean differences (SMD) and variance ratios, which quantify between-group differences in means and dispersion. All covariates showed SMDs below 0.23 and variance ratios between 0.55 and 1.55, confirming excellent baseline comparability.

### Visit selection and temporal alignment

Only ON-medication (for DBS after surgery, ON-medication + ON-stimulation) visits were analyzed. Because PPMI visit schedules were non-standardized, a reference visit was defined for each DBS patient as the last preoperative visit. The most similar non-DBS reference visit was then selected, and follow-up visits were included if their interval from the reference visit differed by no more than 180 days between matched pairs resulting in 40 matched pairs with complete data sets. This number decreased at later timepoints (34 and 18 for the first and second follow-ups, respectively); subsequent timepoints were excluded because of small sample sizes. This reduction reflected variable visit schedules and incomplete longitudinal data rather than systematic dropout or selection bias (Table 1).

**Table 1:** Timing of study visits

Timepoint	*n* per group	Time relative to surgery [years]; mean (SD); (DBS group)	Time relative to reference visit [years]; mean (SD); (DBS group)	Time relative to reference visit [years]; mean (SD); (non-DBS group)
1	40	−0.85 (0.74)	0.00 (0.00)	0.00 (0.00)
2	34	0.93 (0.95)	1.74 (1.11)	1.71 (1.08)
3	18	2.06 (1.16)	2.91 (1.26)	2.89 (1.28)
4	7	3.84 (2.09)	4.48 (2.13)	4.44 (2.13)

Timing of study visits relative to surgery or reference visit in the DBS and non-DBS groups. Values are number of participants (*n*) and mean (SD) follow-up interval in years for each group. Timepoints are listed relative to the surgical date for DBS patients and to a matched reference visit for both groups.

### Baseline characteristics

Mean disease duration was 8.76 years in the DBS group and 9.08 years in the non-DBS group (SMD = −0.11), mean age was 60.85 vs. 62.91 years (SMD = −0.23). MDS-UPDRS scores were I (rater: 1.95 vs. 2.28, SMD = −0.14), I (patient: 7.98 vs. 8.75, SMD = −0.17), II (12.85 vs. 13.03, SMD = −0.03), III (21.08 vs.21.35, SMD = −0.02), and IV (7.48 vs. 7.18, SMD = −0.07). Temporal alignment remained excellent across follow-up intervals (for example, timepoint 2: DBS 1.74 ± 1.11 years, non-DBS 1.71 ± 1.08 years; timepoint 3: DBS 2.91 ± 1.26 years, non-DBS 2.89 ± 1.28 years).

### Outcome measures

**Fig. 1:**
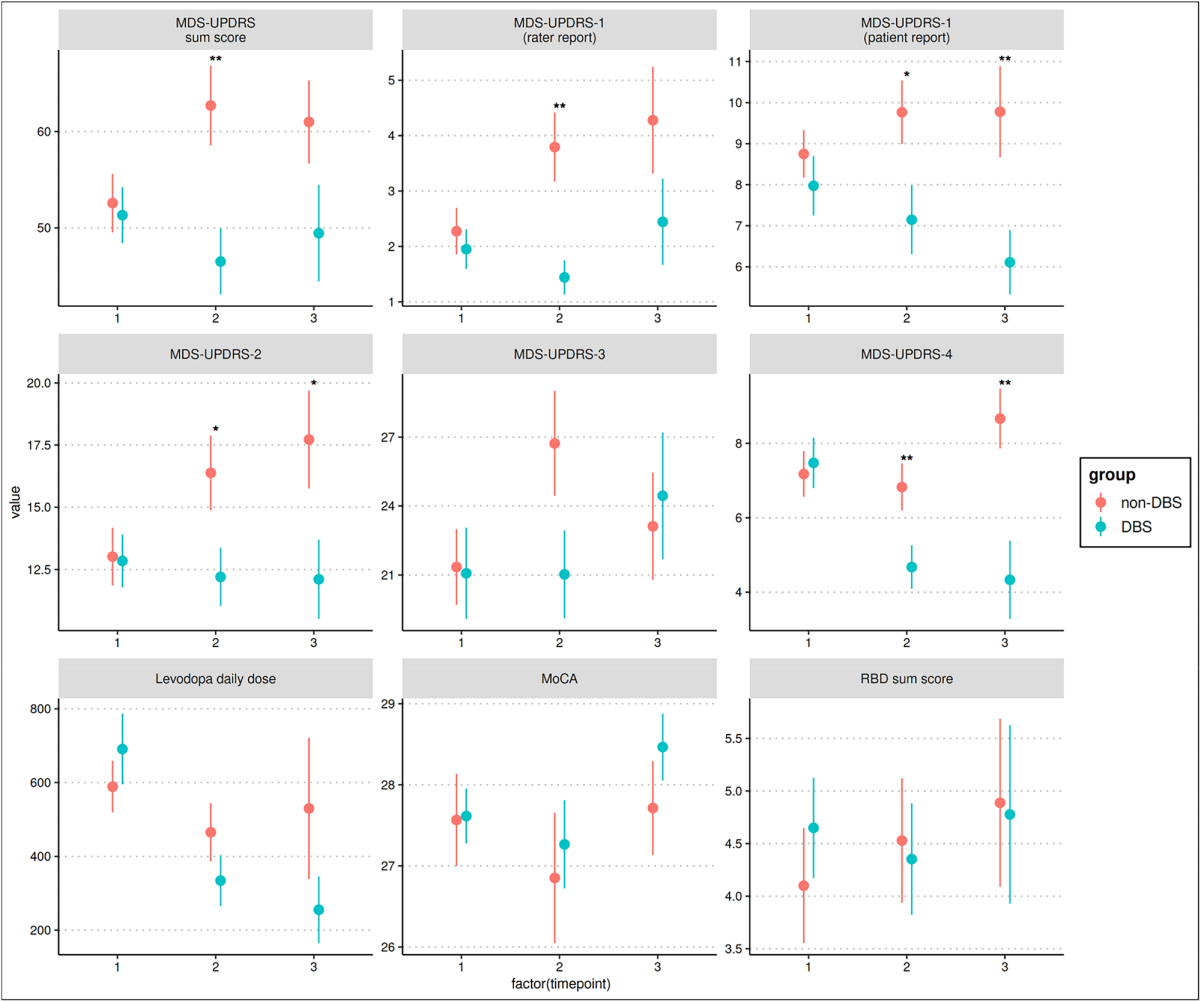
Changes in clinical outcomes by group over time. Mean values with standard deviations are shown for each outcome at baseline (timepoint 1), 1.7 years (timepoint 2), and 2.9 years (timepoint 3) after baseline. Measures include MDS-UPDRS total and subscales (clinician and patient-rated), levodopa equivalent daily dose (LEDD), Montreal Cognitive Assessment (MoCA), and REM sleep behavior disorder (RBDQ) sum score. Red markers represent non-DBS patients and blue markers represent DBS patients. Asterisks indicate statistically significant between group differences (*p < 0.05, **p < 0.01).

Primary outcomes included MDS-UPDRS total and subscale scores (Parts I–IV), Montreal Cognitive Assessment (MoCA), and REM Sleep Behavior Disorder Questionnaire (RBDQ) sum score. Group comparisons across all timepoints are summarized in Table 2 and Fig. 1. At baseline, the matched DBS and non-DBS groups did not differ in age, disease duration, total MDS-UPDRS scores or any subscale score. MoCa, RRBDQ, and LEDD were also comparable, confirming robust baseline balance after matching.

**Table 2:** Longitudinal changes in clinical outcomes

	Baseline (timep. 1)	1.7 years (timepoint 2)	2.9 years (timep. 3)
MDS-UPDRS_SUM	*t* = −0.335, *p* = 0.739, diff = −1.250, CI_diff = [−8.794, 6.294], *d* = −0.075	*t* = −3.015, *p* = 0.004 **, diff = −16.206, CI_diff = [−26.937, −5.475], *d* = −0.731	*t* = −1.944, *p* = 0.069, diff = −11.556, CI_diff = [−24.095, 0.984], *d* = −0.648
MDS-UPDRS 1 rater	*t* = −0.591, *p* = 0.556, diff = −0.325, CI_diff = [−1.420, 0.770], *d* = −0.132	*t* = −3.450, *p* = 0.002 **, diff = −2.353, CI_diff = [−3.740, −0.966], *d* = −0.837	*t* = −1.943, *p* = 0.069, diff = −1.833, CI_diff = [−3.824, 0.158], *d* = −0.648
MDS-UPDRS 1 patient	*t* = -0.916, *p* = 0.365, diff = −0.775, CI_diff = [−2.487, 0.937], *d* = −0.205	*t* = −2.293, *p* = 0.025 *, diff = −2.618, CI_diff = [−4.897, −0.338], *d* = −0.556	*t* = −2.984, *p* = 0.008 **, diff = −3.667, CI_diff = [−6.259, −1.074], *d* = −0.995
MDS-UPDRS 2	*t* = -0.114, *p* = 0.910, diff = −0.175, CI_diff = [−3.275, 2.925], *d* = −0.026	*t* = -2.346, *p* = 0.025 *, diff = −4.176, CI_diff = [−7.799, −0.554], *d* = -0.569	*t* = −2.214, *p* = 0.034 *, diff = −5.611, CI_diff = [−10.761, −0.461], *d* = −0.738
MDS-UPDRS 3	*t* = −0.111, *p* = 0.912, diff = −0.275, CI_diff = [−5.304, 4.754], *d* = −0.025	*t* = −1.913, *p* = 0.060, diff = −5.698, CI_diff = [−11.647, 0.251], *d* = −0.467	*t* = 0.360, *p* = 0.721, diff = 1.319, CI_diff = [−6.139, 8.778], *d* = 0.124
MDS-UPDRS 4	*t* = 0.887, *p* = 0.380, diff = 0.300, CI_diff = [−0.384, 0.984], *d* = 0.198	*t* = −2.869, *p* = 0.007 **, diff = −2.147, CI_diff = [−3.669, −0.625], *d* = −0.696	*t* = −3.581, *p* = 0.002 **, diff = −4.333, CI_diff = [−6.886, −1.780], *d* = −1.194
LEDD	*t* = 0.862, *p* = 0.391, diff = 101.998, CI_diff = [−133.608, 337.603], *d* = 0.193	*t* = -1.247, *p* = 0.217, diff = −130.979, CI_diff = [−340.617, 78.658], *d* = −0.303	*t* = −1.299, *p* = 0.203, diff = −274.741, CI_diff = [−704.704, 155.222], *d* = −0.433
MoCA	*t* = 0.073, *p* = 0.942, diff = 0.048, CI_diff = [−1.255, 1.350], *d* = 0.017	*t* = 0.435, *p* = 0.665, diff = 0.415, CI_diff = [−1.497, 2.327], *d* = 0.115	*t* = 1.316, *p* = 0.208, diff = 0.796, CI_diff = [−0.496, 2.088], *d* = 0.489
RBDQ	*t* = 0.775, *p* = 0.443, diff = 0.550, CI_diff = [−0.886, 1.986], *d* = 0.173	*t* = −0.275, *p* = 0.785, diff = −0.176, CI_diff = [−1.483, 1.130], *d* = −0.067	*t* = −0.095, *p* = 0.925, diff = −0.111, CI_diff = [−2.483, 2.261], *d* = −0.032

Changes in clinical outcomes over time were evaluated by comparing matched DBS and non-DBS participants at each follow-up time point. For each outcome and each time point, group differences were estimated using linear mixed models with DBS status as a fixed effect and a random intercept for the matched subclass, which represents a mixed effects generalization of a paired design. Outcomes included MDS-UPDRS total score and Parts 1 (clinician and patient rated) to 4, levodopa equivalent daily dose (LEDD), cognitive function (MoCA), and REM sleep behavior disorder score (RBDQ sum score). For each comparison, we report the estimated group difference (DBS minus non-DBS), its 95% confidence interval, the t-statistic, the *p*-value, and Cohen’s *d*. Asterisks denote statistical significance (*p < 0.05, **p < 0.01, ***p < 0.001).

## Supplementary information

Supplementary information

## Data Availability

Data used in the preparation of this article was obtained on 2024-04-25 from the Parkinson’s Progression Markers Initiative (PPMI) database (www.ppmi-info.org/access-data-specimens/download-data), RRID:SCR_006431. For up-to-date information on the study, visit www.ppmi-info.org.

## Code availability

Code supporting the key findings of this study will be made available for researchers upon request after journal publication, in accordance with ethical and legal regulations.
